# Intramural pregnancy after intrauterine insemination in a nulligravid patient without previous uterine trauma, complicated by idiopathic thrombocytopenic purpura: A case report

**DOI:** 10.1016/j.crwh.2025.e00684

**Published:** 2025-01-09

**Authors:** Koyo Yamamoto, Tsuyoshi Takiuchi, Kengo Kiso, Saki Ishii, Satoshi Nakagawa, Yasuto Kinose, Michiko Kodama, Yutaka Ueda, Kenjiro Sawada, Takahiro Tsuboyama, Tadashi Kimura

**Affiliations:** a Department of Obstetrics and Gynecology, Graduate School of Medicine, The University of Osaka, 2-2, Yamadaoka, Suita, Osaka 565-0871, Japan; b Department of Clinical Genomics, Graduate School of Medicine, The University of Osaka, 2-2, Yamadaoka, Suita, Osaka 565-0871, Japan; c Department of Radiology, The University of Osaka, 2-2, Yamadaoka, Suita, Osaka 565-0871, Japan; d Department of Radiology, Kobe University, 7-5-2, Kusunoki-cho, Chuo-ku, Kobe, Hyogo 650-0017, Japan; e Sakai City Medical Center, 1-1-1, Ebaraji-cho, Nishi-ku, Sakai, Osaka 593-8304, Japan

**Keywords:** Intramural pregnancy, Ectopic pregnancy, Intrauterine insemination

## Abstract

Intramural pregnancy (IMP) is an extremely rare form of ectopic pregnancy (EP), typically associated with previous uterine trauma, adenomyosis, or assisted reproductive technology (ART), such as embryo transfer (ET). Despite its potentially life-threatening nature, the absence of definitive preoperative diagnostic criteria for IMP complicates its early detection and management, especially in patients without known risk factors. Additionally, management becomes more challenging when there is an elevated risk of hemorrhage. We report the case of a 34-year-old nulligravid woman referred to a tertiary hospital with suspected EP and bilateral ovarian endometriomas following intrauterine insemination. The patient had no history of uterine trauma or ET. Blood tests and ultrasonography supported the diagnosis of EP, and computed tomography suggested peritoneal pregnancy. Upon further investigation, the patient was diagnosed with idiopathic thrombocytopenic purpura, presenting with a platelet count of 30,000/μL. Due to the associated risk of hemorrhage, we proceeded with emergency exploratory laparoscopy after platelet transfusion. Intraoperatively, when an IMP was identified, the procedure was rapidly converted to laparotomy owing to bleeding risk associated with idiopathic thrombocytopenic purpura. The gestational sac covered with the uterine serosa was dissected, and the uterine defect was repaired to preserve fertility. The blood loss was 320 mL. The patient's postoperative recovery was uneventful, and histopathological examination confirmed the diagnosis of IMP. The patient later resumed ART and successfully achieved term pregnancy, leading to a normal vaginal delivery 3 years after the initial surgery. Early diagnosis and appropriate management of IMP are critical to prevent severe intraperitoneal bleeding, while preserving future fertility.

## Highlights

• Intramural pregnancy (IMP) is a very rare condition with few diagnostic criteria.
• We report a case of IMP after intrauterine insemination in a nulligravid patient without previous uterine trauma.
• The patient was diagnosed with idiopathic thrombocytopenic purpura.
• Timely diagnosis and treatment were crucial for improving the patient outcome.

## Graphical abstract

**Figure:**
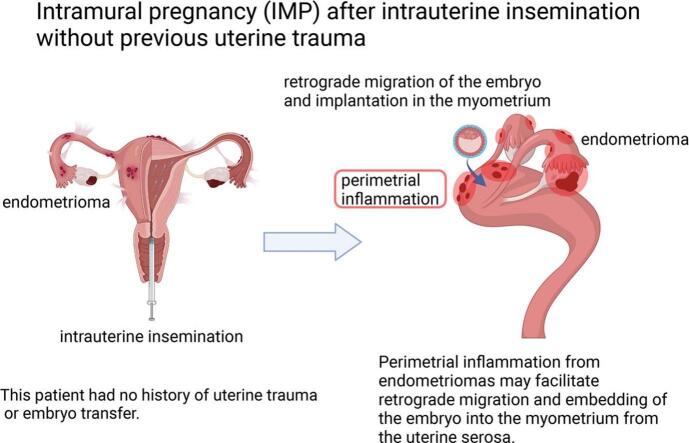

## 1 Introduction

Ectopic pregnancy (EP) represents approximately 1–2 % of all spontaneous pregnancies, with the vast majority occurring in the fallopian tube [1]. Intramural pregnancy (IMP) is a rare form of EP, accounting for less than 1 % of all EP cases, in which gestation occurs within the myometrium without any connection to the fallopian tubes or endometrial cavity [2]. Most IMP cases are associated with a history of uterine trauma, adenomyosis, or the use of assisted reproductive technology (ART), such as embryo transfer (ET). Additionally, perimetrial inflammation has also been implicated in the development of IMP [3]. However, the pathophysiology of IMP remains unclear.

The diagnosis and management of IMP are challenging because cases are scarce and available evidence is limited. IMP typically presents with nonspecific symptoms similar to other forms of EP, including vaginal bleeding, abdominal pain, and amenorrhea [4]. Despite advancements in ultrasonography, early diagnosis of IMP remains difficult, and many cases are diagnosed only intraoperatively [5]. Management selection depends on factors such as the location of conception, gestational age, desire to preserve future fertility, and the patient's condition [6]. Early and appropriate diagnosis and management of IMP are critical, especially in high-risk patients such as those with an increased risk of hemorrhage. Timely intervention can reduce the perioperative risks associated with invasive procedures such as uterine incision, and can facilitate fertility preservation [7].

Herein, we report a case of IMP in a nulligravid patient without a history of uterine trauma after intrauterine insemination (IUI). The patient was diagnosed with idiopathic thrombocytopenic purpura (ITP), necessitating platelet transfusion. The diagnosis of IMP was confirmed intraoperatively and the condition was successfully treated surgically.

## 2 Case Presentation

The patient was a 34-year-old nulligravid woman with suspected bilateral ovarian endometriomas; no obvious evidence of adenomyosis was observed in the uterus by transvaginal ultrasonography. Hysterosalpingography (HSG) demonstrated bilateral tubal patency, although diagnostic laparoscopy was not performed. The patient had no history of uterine trauma and had undergone IUI at another facility. Twenty-seven days later, her serum β-human chorionic gonadotropin (hCG) level was 22,069 mIU/mL; however, transvaginal ultrasound did not reveal an intrauterine gestational sac. Consequently, she was referred to a tertiary hospital with a suspected EP.

**Fig. 1:**
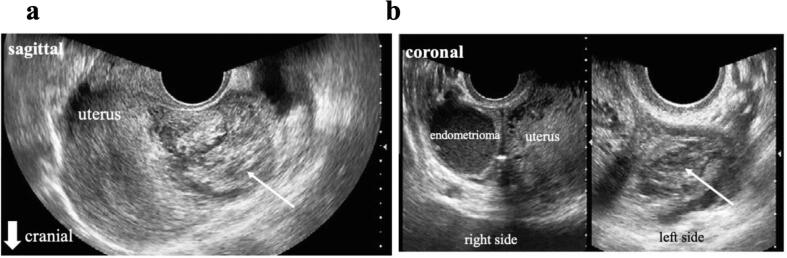
(a) Transvaginal ultrasound indicates an empty uterine cavity with no evidence of a gestational sac, along with the heterogeneous mass (arrow) on the posterior wall of the uterus. (b) Transvaginal ultrasound shows an ovarian endometrioma and the heterogeneous mass (arrow).

**Fig. 2:**
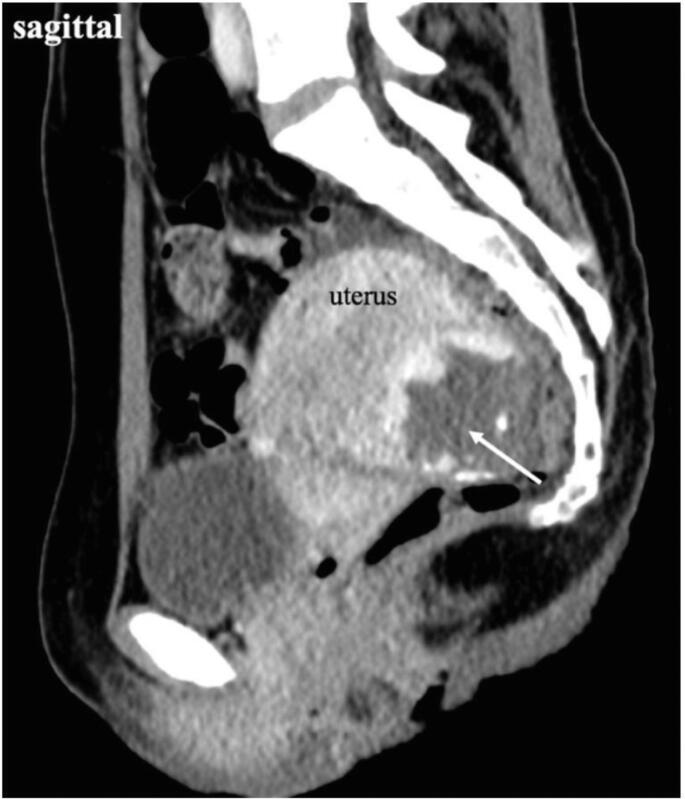
Sagittal contrast-enhanced CT scan of the pelvis demonstrates a mass posterior to the uterus (arrow). The mass shows marked enhancement at the periphery and was suspected to be a gestational sac implanted in the peritoneum.

On physical examination at the tertiary hospital, her vital signs were normal and she had no abdominal pain, or vaginal bleeding. Transvaginal ultrasonography revealed an empty uterine cavity without evidence of a gestational sac, along with a heterogeneous mass on the posterior wall of the uterus (Fig. 1a) and a right ovarian endometrioma and the heterogeneous mass (Fig. 1b). Her initial blood tests were as follows: hemoglobin level 13.7 g/dL, white blood cell count 7070/μL, and a platelet count 30,000/μL. Her serum β-hCG level was 24,278 mIU/mL. After consultation with a hematologist, the patient was diagnosed with ITP. Contrast-enhanced computed tomography suggested peritoneal pregnancy (Fig. 2). Considering the risk of hemorrhage due to ITP, a decision was made to proceed with emergency exploratory laparoscopy after platelet transfusion.

**Fig. 3:**
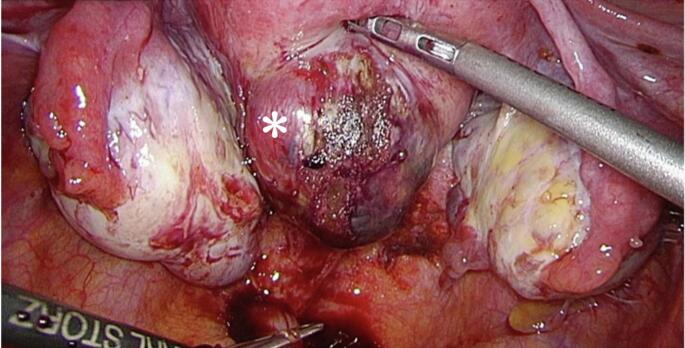
Laparoscopic intraoperative views show the bulging mass (*) measuring 3 cm in the posterior wall of the uterus with a dark red surface. (For interpretation of the references to colour in this figure legend, the reader is referred to the web version of this article.)

During laparoscopy, a small amount of blood was present in the pelvic cavity along with superficial peritoneal endometriosis. Both fallopian tubes appeared normal, and the ovaries were enlarged to 3–4 cm owing to endometriomas. An approximately 3 cm bulging mass with a dark red surface was observed on the posterior wall of the uterus (Fig. 3). The mass was not connected to the fallopian tubes or peritoneum. The overlying serosa was compressed and thin but not ruptured. However, the bulging mass ruptured during the procedure, leading to bleeding. Dark red tissue from the mass was exposed, and careful exploration confirmed that it was not connected to the endometrial cavity. Owing to the risk of bleeding associated with ITP, a rapid conversion from laparoscopy to laparotomy was performed. The mass was excised, and the uterine defect was repaired and sutured. Subsequently, a curettage was performed intraoperatively under direct visualization to ensure safety. The operation lasted 141 min, with blood loss of 320 mL.

**Fig. 4:**
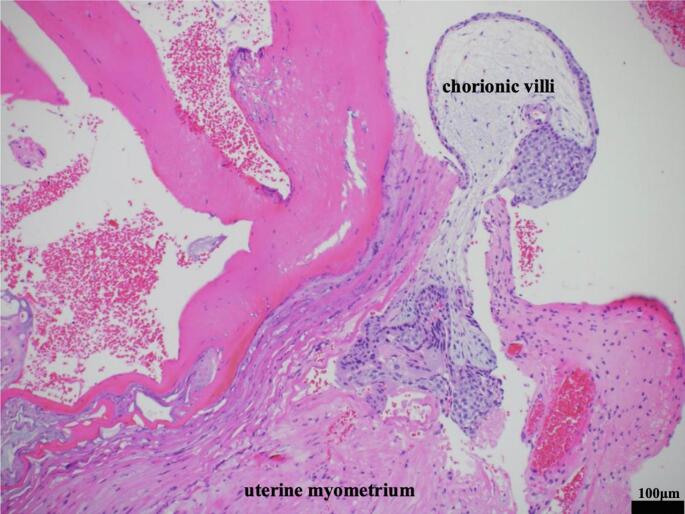
Histopathological examination shows the presence of chorionic villi in the uterine myometrium (hematoxylin and eosin, original magnification ×200).

Histopathological examination of the excised tissue revealed chorionic villi within the uterine myometrium, with trophoblast cells infiltrating the muscle tissue and the absence of decidual tissue, confirming the diagnosis of IMP (Fig. 4). Histopathological examination of the curettage specimen revealed only decidual tissue, with no presence of chorionic villi.

The patient's serum β-HCG level declined to 8899 mIU/mL on the first postoperative day. The postoperative course was uneventful, and she was discharged in good condition. Hysteroscopy and HSG performed three months after surgery confirmed that there were no abnormalities in the uterine cavity and no sinuses connecting the cavity to the myometrium. Later, the patient successfully conceived through ART and carried the pregnancy to full term, delivering vaginally 3 years after the initial surgery.

## 3 Discussion

IMP is a rare form of EP, in which pregnancy occurs within the myometrium without any connection to the fallopian tubes, round ligament, or endometrial cavity. The condition was first reported by Doderlein and Herzog in 1913 [8], and accounts for less than 1 % of all EP [9]. Common symptoms of IMP include vaginal bleeding, abdominal pain, and amenorrhea, similar to those of other forms of EP [4]. The most frequent location of the gestational sac in IMP is the posterior uterine wall, particularly the left fundus [3]. In the present case, although the patient was asymptomatic, the disease could be diagnosed and managed at an early gestational age because she was undergoing infertility treatment. During surgery, the implantation site was observed in the posterior wall of the uterus, which is consistent with the most common location.

Various risk factors have been proposed for IMP, including uterine trauma from surgical procedures, such as cesarean section, myomectomy, salpingectomy, dilation and curettage, ET, and adenomyosis [10]. With the global increase in ART use, the prevalence of IMP is expected to increase, and obstetricians and gynecologists should be aware of this condition. It has been hypothesized that these factors contribute to the formation of microscopic sinus tracts within the endometrium, allowing the conceptus to enter the myometrium [11]. In ART, the ET catheter may inadvertently penetrate the endometrium or dilate the sinus tracts [7]. Additionally, in patients with adenomyosis, the embryo may also implant into the myometrium due to serosal inflammation and dysfunction of the deep adenomyosis, which contains sufficient endometrial tissue that responds to estrogen and progesterone, leading to decidualization and potentially providing a site for blastocyst implantation [12]. Moreover, perimetrial inflammation from endometriosis may facilitate retrograde migration and embedding of the embryo into the myometrium from the uterine serosa [3]. In this patient, it was suspected that perimetrial inflammation caused by bilateral ovarian endometriomas contributed to the retrograde migration of the embryo and its implantation in the myometrium. Notably, no evidence of adenomyosis was observed in this patient, either histopathologically in the excised tissue or through postoperative imaging findings such as asymmetric myometrial thickening or myometrial cyst on transvaginal ultrasonography. Also, as demonstrated in this case, investigations recommended prior to performing IUI, such as HSG, might be considered as a potential source of uterine trauma that may increase the risk of IMP. In a recent systematic review by Ntafam et al. [10], only one of 82 cases of IMP involved IUI as the mode of conception (which was originally reported by Kumtepe et al. [12]) and involved no prior uterine trauma or adenomyosis. In addition, 73 of the 82 IMP cases had had prior pregnancies, suggesting that a history of pregnancy may also be considered a risk factor for IMP [10]. However, unlike Kumtepe et al.'s case, in which the patient experienced a prior miscarriage, the patient in the present report developed IMP during her first pregnancy, exemplifying the rarity of her case.

The management of IMP is determined by factors such as the location of conception, the patient's condition, gestational age, and desire to preserve fertility [2,13]. Treatment options include expectant management, medical treatment (*e.g.*, methotrexate), and surgical intervention. Surgical treatments for IMP range from enucleation to hysterectomy, depending on the severity of the condition. Achieving adequate bleeding control while preserving fertility is critical, as minimizing unnecessary tissue trauma and avoiding excessive suturing can help preserve uterine function [14]. In the present case, a preoperative diagnosis of IMP was not possible, and peritoneal pregnancy was initially suspected. However, because of the patient's concurrent ITP diagnosis, expectant management was considered to pose a significant hemorrhagic risk. Intraoperatively, IMP was unexpectedly diagnosed; however, the decision to proceed with surgery was justified due to the high risk of bleeding associated with ITP. After sufficient platelet transfusion and confirmation of platelet recovery, it was determined that the patient could tolerate the uterine myometrial incision required for an unexpected diagnosis of IMP, and the lesion was successfully excised. Subsequently, a curettage was performed intraoperatively under direct visualization to definitively exclude an intrauterine abnormal pregnancy, as the findings at that time did not provide absolute certainty of IMP. However, it is acknowledged that curettage may not have been absolutely necessary in this case. If performed, curettage should be conducted with utmost caution and under direct visualization to ensure safety and to avoid complications, such as damaging the repair in the myometrium. As surgical procedures can affect uterine invasion, it is crucial to provide preoperative explanations considering the possibility of IMP. Without an accurate diagnosis and timely treatment, IMP can result in uterine rupture, massive intraperitoneal bleeding, and even death. Despite the challenges in the preoperative diagnosis of this case, the condition was successfully managed surgically, considering the high risk of massive bleeding due to ITP.

The management of infertility in women with endometriomas presents a complex clinical challenge that necessitates individualized treatment decisions. While the guidelines from the UK National Institute for Health and Care Excellence (NICE) recommend surgical excision of endometriomas followed by *in vitro* fertilization (IVF) as the preferred approach, alternative treatments such as ovulation induction with IUI are considered viable under specific circumstances [15]. For example, the American Society for Reproductive Medicine supports ovulation induction with IUI or IVF in cases of advanced maternal age [16], and the guidelines from the European Society of Human Reproduction and Embryology indicate that in patients with revised American Society for Reproductive Medicine stage III/IV endometriosis and patent fallopian tubes, ovulation induction with IUI remains a consideration [17]. In the present case, the patient's HSG confirmed bilateral tubal patency, and her ovarian reserve was normal, leading to the initial decision to pursue IUI despite the absence of diagnostic laparoscopy or surgical intervention. While this approach deviates from the conventional pathway outlined by NICE, it reflects a clinical judgment tailored to the patient's unique circumstances and preferences. However, it is important to recognize that HSG and other diagnostic procedures can potentially cause uterine trauma, which may contribute to the risk of IMP. This underscores the need for clinicians to carefully weigh the benefits and risks of various diagnostic and treatment strategies when managing similar cases.

## 4 Conclusion

We present the case of a 34-year-old nulligravid patient with IMP following IUI, without any history of uterine trauma. Despite the intraoperative diagnosis, the surgical treatment was successful, particularly in managing the hemorrhage risk associated with ITP. Although IMP is rare, it can have life-threatening consequences, underscoring the importance of early diagnosis and management to improve patient outcomes.

## References

[1] Bernstein H.B., Thrall M.M., Clark W.B. (2001). Expectant management of intramural ectopic pregnancy. Obstet. Gynaecol..

[2] Ong C. (2010). Sonographic diagnosis and successful medical management of an intramural ectopic pregnancy. J. Clin. Ultrasound.

[3] Zhang Q. (2019). Intramural ectopic pregnancy following pelvic adhesion: case report and literature review. Arch. Gynecol. Obstet..

[4] Liu N. (2017). Ultrasound diagnosis of intramural pregnancy. J. Obstet. Gynaecol. Res..

[5] Kirk E. (2013). Intramural ectopic pregnancy: a case and review of the literature. Eur. J. Obstet. Gynecol. Reprod. Biol..

[6] Harris R. (2024). From intramural ectopic pregnancy to hysterectomy: a case report. Case Rep. Womens Health.

[7] Liu Y., Wu Y. (2020). Intramyometrial pregnancy after cryopreserved embryo transfer: a case report. BMC Pregnancy Childbirth.

[8] Doederlein T.O., Herzog M. (1913). A new type of ectopic gestation: pregnancy in an adenomyoma uteri. Surg Gynecol Obstet.

[9] Glass T. (2010). Intramural pregnancy presenting in a patient with tuberous sclerosis. J. Clin. Ultrasound.

[10] Ntafam C.N., Sanusi-Musa I., Harris R.D. (2023). Intramural ectopic pregnancy: an individual patient data systematic review. Eur. J. Obstet. Gynecol. Reprod. Biol. X.

[11] Vagg D. (2018). Intramural ectopic pregnancy following myomectomy. J. Investig. Med. High Impact Case Rep..

[12] Kumtepe Y. (2007). The rarest form of ectopic pregnancy: intramural ectopic pregnancy and medical treatment. J. Turk. Ger. Gynecol. Assoc..

[13] Memtsa M. (2013). Diagnosis and management of intramural ectopic pregnancy. Ultrasound Obstet. Gynecol..

[14] Ng S. (2009). Laparoscopic management of 53 cases of cornual ectopic pregnancy. Fertil. Steril..

[15] National Institute for Health and Care Excellence (NICE) (2017). https://www.nice.org.uk/guidance/ng73.

[16] American Society for Reproductive Medicine (ASRM) (2012). https://www.asrm.org/globalassets/_asrm/practice-guidance/practice-guidelines/pdf/endometriosis_and_infertility.pdf.

[17] European Society of Human Reproduction and Embryology (ESHRE) (2022). https://www.eshre.eu/Guidelines-and-Legal/Guidelines/Endometriosis-guideline.

